# Cholesterol in colorectal cancer: an essential but tumorigenic precursor?

**DOI:** 10.3389/fonc.2023.1276654

**Published:** 2023-11-03

**Authors:** Xing He, Huanrong Lan, Ketao Jin, Fanlong Liu

**Affiliations:** ^1^ Department of Gastroenterology, Jinhua Wenrong Hospital, Jinhua, Zhejiang, China; ^2^ Department of Surgical Oncology, Hangzhou Cancer Hospital, Hangzhou, Zhejiang, China; ^3^ Department of Colorectal Surgery, Affiliated Jinhua Hospital, Zhejiang University School of Medicine, Jinhua, Zhejiang, China; ^4^ Department of Colorectal Surgery, The First Affiliated Hospital, Zhejiang University School of Medicine, Hangzhou, Zhejiang, China

**Keywords:** cholesterol, metabolism, colorectal cancer, tumorigenic, therapeutic approaches

## Abstract

Colorectal cancer (CRC) is one of the most lethal human malignancies, and with the growth of societies and lifestyle changes, the rate of people suffering from it increases yearly. Important factors such as genetics, family history, nutrition, lifestyle, smoking, and alcohol can play a significant role in increasing susceptibility to this cancer. On the other hand, the metabolism of several macromolecules is also involved in the fate of tumors and immune cells. The evidence discloses that cholesterol and its metabolism can play a role in the pathogenesis of several cancers because there appears to be an association between cholesterol levels and CRC, and cholesterol-lowering drugs may reduce the risk. Furthermore, changes or mutations of some involved genes in cholesterol metabolism, such as CYP7A1 as well as signaling pathways, such as mitogen-activated protein kinase (MAPK), can play a role in CRC pathogenesis. This review summarized and discussed the role of cholesterol in the pathogenesis of CRC as well as available cholesterol-related therapeutic approaches in CRC.

## 1 Introduction

Colorectal cancer (CRC) is the third most common cancer and the second cause of cancer-related deaths. Many people, particularly those over 50 years, are affected by CRC yearly (1). Generally, the proliferation of epithelial cells in normal colon and GI is dysregulated in patients with CRC (2). The most frequent cause of death in CRC patients is metastasis of tumor cells to the liver, bones, lungs, brain, and spinal cord (3). In addition to people over 50, regarding genetic and family history, others with various age ranges could be at risk for CRC (4, 5). The most common risk factors in CRC are heredity, family history, gene mutations, GI microbiome pattern, obesity, smoking, alcoholism, and poor nutrition (low-fiber and high-fat diets). Furthermore, human disorders, such as inflammatory bowel disease (IBD), gastrointestinal adenomatous polyps, and diabetes, are considered other CRC risk factors (2). Polyps, as small and nontumoral masses that could be detected in the large intestine, are the fundamental core of CRC. It has been revealed that, upon chronic inflammation, polyps may ultimately turn into tumoral masses (6, 7). CRC is the third most frequently diagnosed type of malignancy, and targeted therapies have been ineffective for cases with RAF or RAS mutations (8).

Cholesterol plays an essential role in cell physiology (9). Steroid hormones, vitamin D, and bile acids are synthesized from cholesterol, a structural component of cell membranes. Furthermore, cholesterol is involved in regulating the function of cells and its structural function in providing stability and fluidity (10). The findings of preclinical and clinical studies revealed that high-fat diets and hypercholesterolemia are involved in tumorigenesis and cancer development through activating various metabolic pathways. Exogenous cholesterol activates the oncogenic Hedgehog pathway, and the mammalian target of rapamycin complex 1 (mTORC1) can be activated by endogenous cholesterol. Lipid rafts are the most critical signaling platforms for tumor cells. Therefore, cholesterol as a central component of lipid rafts could contribute to cancer progression (9). Studies reported that cholesterol could participate in CRC development (11). Based on previous investigations, serum cholesterol levels significantly increased in CRC patients (12).

Besides, cholesterol-rich foods can alter blood lipids’ patterns and induce bile acid formation in the liver. The excretion of bile acids in the bile may stimulate Single Nucleotide Polymorphisms (SNPs) in *CYP7A1*. Previously, it was reported that SNPs in CYP7A1, the rate-limiting enzyme in the metabolic pathway responsible for cholesterol converting to primary bile acids, were associated with an increased risk of CRC (13). Furthermore, low-density lipids (LDLs), by activating the mitogen-activated protein kinase (MAPK) pathway and the production of reactive oxygen species (ROS), promote intestinal inflammation and CRC development (14). Accordingly, targeting cholesterol and its metabolic pathways could be considered a potential treatment in CRC patients. For instance, Bisphosphonate and statin therapies modulate the cellular cholesterol biosynthesis pathway and reduce the prenylation of G-proteins, inhibiting tumor cell growth pathways involved in CRC. Chelating membrane cholesterol is another effective anticancer approach that interrupts lipid raft functions. According to the available data, proprotein convertase Subtilisin/Kexin type 9 (PSCK9) induces adenomatous polyposis colitis (*APC*)/*KRAS*-mutant CRC. In addition, *de novo* cholesterol biosynthesis can promote *APC/KRAS* mutant CRC, along with high levels of an essential metabolite for KRAS activation, termed geranylgeranyl diphosphate (GGPP) (15). Accordingly, PSCK9 could be a therapeutic target in CRC.

This review summarized the role of cholesterol in the pathogenesis of CRC and discussed related therapeutic tactics and the advantages and disadvantages of these therapies.

## 2 Biology of cholesterol

Cholesterol is an essential molecule in the body. It plays a polygonal role in maintaining cellular homeostasis and overall health. Cholesterol is primarily known for its presence in cell membranes, where it modulates permeability and fluidity, guaranteeing structural integrity and proper cell functioning (16). Additionally, cholesterol plays a vital role in serving as a precursor for synthesizing crucial molecules, including sex hormones like testosterone and estrogen and steroid hormones such as aldosterone and cortisol. Additionally, it plays a key role in producing bile acids, essential for digestion and absorption of dietary fats. Cholesterol is transported in the bloodstream via lipoproteins, notably LDL and HDL, with LDL often referred to as “bad” cholesterol due to its association with cardiovascular diseases (CVDs) (17). Therefore, cholesterol’s biological function is maintaining cellular homeostasis and regulating myriad physiological processes indispensable for human health (18, 19).

### 2.1 Biostructure of cholesterol

**Figure 1:**
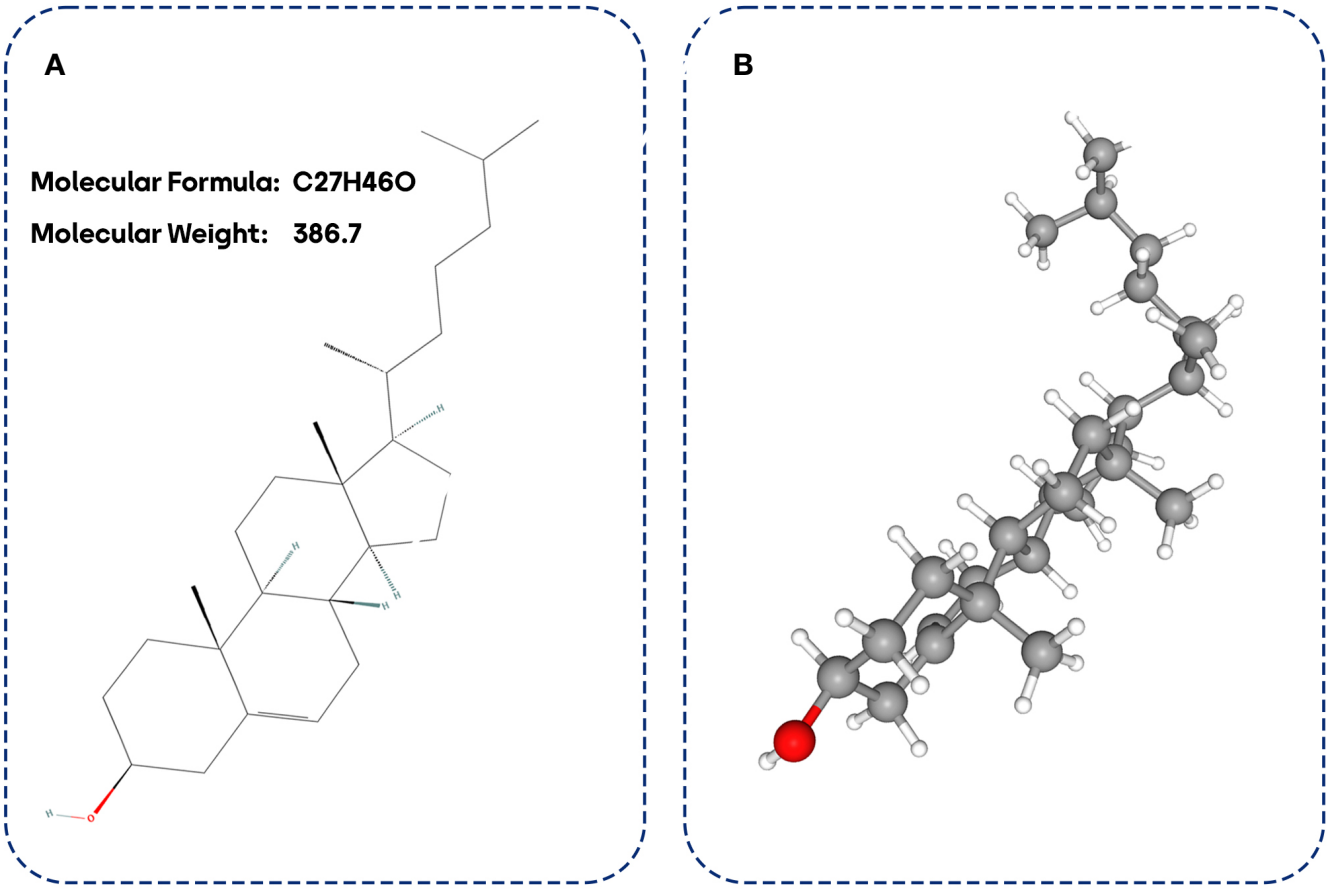
Cholesterol biostructure. 2D cholesterol biostructure **(A)** and 3D cholesterol biostructure **(B)**.

The structure of cholesterol is unique, comprising 27 carbons with a hydrocarbon tail, four hydrocarbon rings in the nucleus, and a hydroxyl group on the side (**Figure 1**). All steroid hormones have this central sterol nucleus or ring. The cholesterol structure’s central ring and hydrocarbon tail are non-polar and not water-soluble. Accordingly, cholesterol (lipid) should be bound to apoproteins (protein) and formed lipoproteins for transportation in blood circulation (10). Besides synthesizing cholesterol *de novo* (10% of total) in the intestines and liver, humans can also obtain it through diet. Exogenous cholesterol and triglycerides are packaged with Apo proteins in the liver before relapsing into the blood circulation as very low-density lipoproteins (VLDL). Evidence has demonstrated that VLDLs contain cholesterol, triglycerides, and phospholipids. When the triglycerides within VLDLs are broken down, cholesterol-rich low-density lipoproteins (LDLs) are formed. These LDLs circulate through the bloodstream and are transported to cells in peripheral tissues that express LDL receptors, where they undergo endocytosis (20, 21). In contrast, high-density lipoproteins (HDLs) carry cholesterol from the peripheral tissues to the liver to reduce cholesterol levels (22).

### 2.2 Biosynthesis of cholesterol

**Figure 2:**
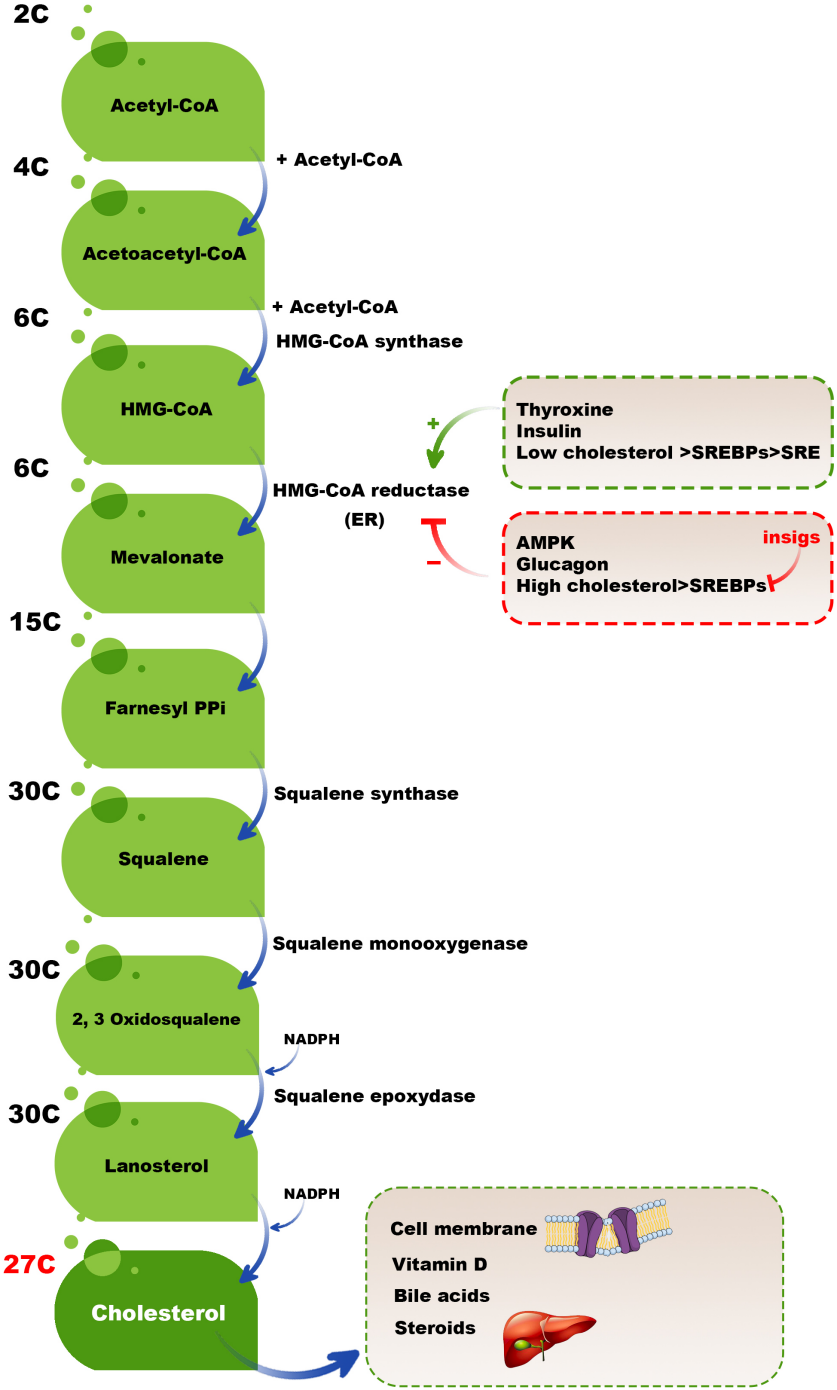
Cholesterol biosynthesis pathway. Two acetyl-CoA molecules generate an acetoacetyl-CoA, and acetyl-CoA third molecule is added to it by activated HMG-CoA synthase to create a six-carbon molecule, HMG-CoA. The next rate-limiting/committed step in cholesterol synthesis is converting HMG-CoA to mevalonate catalyzed by HMG-CoA reductase. After this step, mevalonate is converted into 3-isopentenyl pyrophosphate, farnesyl pyrophosphate, squalene, lanosterol, and finally, cholesterol in several steps.

Although a broad range of cells synthesizes cholesterol, hepatocytes in the liver are the leading site for cholesterol biosynthesis. Firstly, two acetyl coenzyme A (acetyl-CoA) molecules generate an acetoacetyl-CoA and acetyl-CoA third molecule added to it by activated hydroxymethylglutaryl-CoA (HMG-CoA) synthase to create a six-carbon molecule termed 3-hydroxy-3-methyl glutaryl coenzyme A (HMG-CoA). The next rate-limiting/committed step in cholesterol synthesis is converting HMG-CoA to mevalonate catalyzed by HMG-CoA reductase. After this step, mevalonate is converted into 3-isopentenyl pyrophosphate, farnesyl pyrophosphate, squalene, lanosterol, and cholesterol in several steps (**Figure 2**) (23).

High cholesterol levels should be balanced by different mechanisms, such as reducing the activation of HMG-CoA reductase. Adenosine monophosphate (AMP)-activated protein kinase (AMPK) inactivates HMG CoA reductase by phosphorylation of this enzyme because HMG CoA reductase is active in the dephosphorylated state and inactive in the phosphorylated state. In high cholesterol available conditions, some hormones, including thyroxine and insulin, activate HMG CoA reductase; however, glucagon has a reverse mission by inhibiting insulin, activating HMG CoA reductase (24, 25). Moreover, sterol regulatory binding proteins (SREBPs) expressed in the membranes of ER are involved in transcriptional regulating HMG CoA reductase in a dual-role manner. When the cholesterol level is low, the SREBPs are carried to the Golgi complex to process and release an active fragment that can enter into the nucleus and bind to the sterol regulatory element (SRE), upregulating the expression of involved genes in encoding HMG-CoA reductase and also other essential proteins and enzymes cholesterol synthesis. In contrast, insulin-induced gene (INSIG) proteins do not allow SREBPs to transport to the Golgi complex in high cholesterol levels. Therefore, the active fragment of SREBPs cannot release and bind the SRE and activate the expression of HMG-CoA reductase and other essential cholesterol synthesis-mediated enzymes (26) (**Figure 2**). It has been revealed that INSIG1 negatively regulates cholesterol biosynthesis via suppressing *de novo* cholesterol biosynthetic gene expression (27).

## 3 Cholesterol in lipid rafts and aberrant cellular Signaling

Cholesterol plays a fundamental role in the cell membrane’s organization and functions, mainly in specialized microdomains known as lipid rafts. These cholesterol-rich regions are characterized by their higher lipid order and serve as platforms for various physiological processes, such as cell signaling. However, an irregular upsurge in cholesterol levels within lipid rafts can disrupt normal cellular signaling, initiating various pathological states, including cancer and CVDs (28). Moreover, cholesterol is critical for the proper membrane receptor function, including receptor tyrosine kinases (RTKs) and G protein-coupled receptors (GPCRs), which are essential for cell signaling (29). Increased cholesterol levels within lipid rafts can modify receptor conformation and distribution, dysregulating cell-mediated signals. For instance, augmented cholesterol can stimulate constitutive activation of receptor signaling pathways, contributing to abandoned cell growth and proliferation (30). Furthermore, this can lead to the aberrant activation of downstream signaling pathways, such as the phosphoinositide 3-kinase (PI3K)/AKT and MAPK pathways, commonly involved in cancer growth and progression (31). Uncontrolled signaling through these pathways can induce tumor cell survival, proliferation, and metastasis. On the other hand, the accumulation of cholesterol in immune cells-lipid rafts can impair immune responses in the TME, reprogramming tumor milieu (32, 33).

## 4 Cholesterol metabolism in physiologic and pathologic states

Despite the beneficial roles of cholesterol, abnormal cholesterol metabolism can lead to the accumulation of cholesterol in the bloodstream and tissues, increasing the risk of CVDs and other disorders. This section explores the metabolism of cholesterol in both physiologic and pathologic states.

### 4.1 Physiologic states of cholesterol metabolism

Cholesterol metabolism begins with dietary cholesterol intake, primarily found in animal-based foods (34). In the small intestine, cholesterol is absorbed into the enterocytes, the cells that line the gut (35). Dietary cholesterol is incorporated into chylomicrons, lipoprotein particles that transport lipids through the lymphatic system and into the bloodstream (36). The liver is a central organ in cholesterol metabolism. It synthesizes cholesterol *de novo* through a series of enzymatic reactions. A family of transcription factors, such as the SREBPs, tightly regulates this endogenous synthesis (37). When cellular cholesterol levels are low, SREBPs are activated, leading to increased cholesterol synthesis and uptake from the blood. Conversely, when cellular cholesterol levels are high, the liver decreases cholesterol synthesis and increases the synthesis of bile acids (38). Cholesterol is transported through the bloodstream in lipoprotein particles, including LDL and HDL (39). LDL delivers cholesterol to peripheral tissues, while HDL helps to remove excess cholesterol from these tissues and transport it back to the liver in a process known as reverse cholesterol transport (40).

### 4.2 Cholesterol metabolism in pathologic states

#### 4.2.1 Change of cholesterol metabolism in cancer

Cancer is a complex and multifaceted disease characterized by uncontrolled cell growth and division (41). Emerging research suggests that cancer cells often undergo significant changes in cholesterol metabolism, which can impact the progression and characteristics of the disease (9). Cancer cells frequently exhibit an enhanced ability to take up cholesterol from their microenvironment (42). This elevated cholesterol uptake is primarily mediated by the upregulated expression of LDL receptors, scavenger receptor class B type I (SR-B1), and other cholesterol transporters (43). This adaptation allows cancer cells to meet the heightened demand for cholesterol, which is essential for their rapid proliferation and membrane synthesis. In addition to increased cholesterol uptake, many cancer cells boost their endogenous cholesterol production (44). This rise in *de novo* cholesterol synthesis occurs due to the overexpression of key enzymes and transcription factors involved in the cholesterol biosynthetic pathway. Notably, the SREBPs are often upregulated in cancer, promoting the transcription of genes responsible for cholesterol biosynthesis (45). Lipid rafts are specialized cholesterol-rich microdomains in the cell membrane that play a role in signal transduction and cell adhesion. In cancer, alterations in lipid rafts are frequently observed. These changes affect the localization of important signaling proteins, leading to dysregulation of cell growth and survival pathways (46, 47). Elevated cholesterol metabolism in cancer cells has been associated with tumor progression and aggressiveness. High cholesterol levels within the TME can promote angiogenesis and enhance the resistance of cancer cells to apoptosis (48). Moreover, cholesterol can support invadopodia formation, which are cell protrusions that facilitate the invasion of cancer cells into surrounding tissues (49). In CRC, it has been reported that LDL is involved in exacerbating intestinal inflammation and promoting the progression of CRC. LDL is identified as a contributor to these processes, mainly through activating ROS and signaling pathways, including the MAPK pathway. Notably, inflammation has long been recognized as a pivotal factor in cancer initiation and development, and LDL’s role in inciting intestinal inflammation underscores its potential impact on the tumorigenicity of the intestine (14).

Given the association between altered cholesterol metabolism and cancer progression, targeting cholesterol-related pathways has emerged as a potential therapeutic strategy. Several drugs that inhibit cholesterol biosynthesis, such as statins, have shown promise in preclinical studies and early clinical trials for various cancer types (50). Additionally, research into developing cholesterol transporter inhibitors is ongoing (51).

#### 4.2.2 Other pathologic conditions

Hypercholesterolemia is a pathological condition characterized by elevated cholesterol levels in the bloodstream (52). It can result from genetic factors (familial hypercholesterolemia) or unhealthy lifestyle choices, such as a high-fat diet, lack of physical activity, and smoking (53). Elevated LDL cholesterol, often called “bad” cholesterol, is a major risk factor for atherosclerosis and coronary artery disease (54). Atherosclerosis is a pathologic condition that occurs when cholesterol, specifically LDL cholesterol, accumulates within the walls of arteries. This accumulation can lead to the formation of plaques, which narrow the arteries and can ultimately obstruct blood flow (54, 55). If a plaque ruptures, it can trigger the formation of blood clots, potentially leading to heart attacks and strokes (56). Xanthomas are lipid deposits that can form in the skin, tendons, or other soft tissues, primarily due to the accumulation of cholesterol (57). They are associated with familial hypercholesterolemia and can indicate abnormal cholesterol metabolism (58). In addition, cholelithiasis, or the formation of gallstones, is often related to cholesterol metabolism (59). Cholesterol can precipitate out of bile and form solid particles, which may aggregate into gallstones in the gallbladder. This condition can lead to symptoms like pain and, in severe cases, complications requiring surgery.

## 5 Cholesterol and colorectal cancer

**Figure 3:**
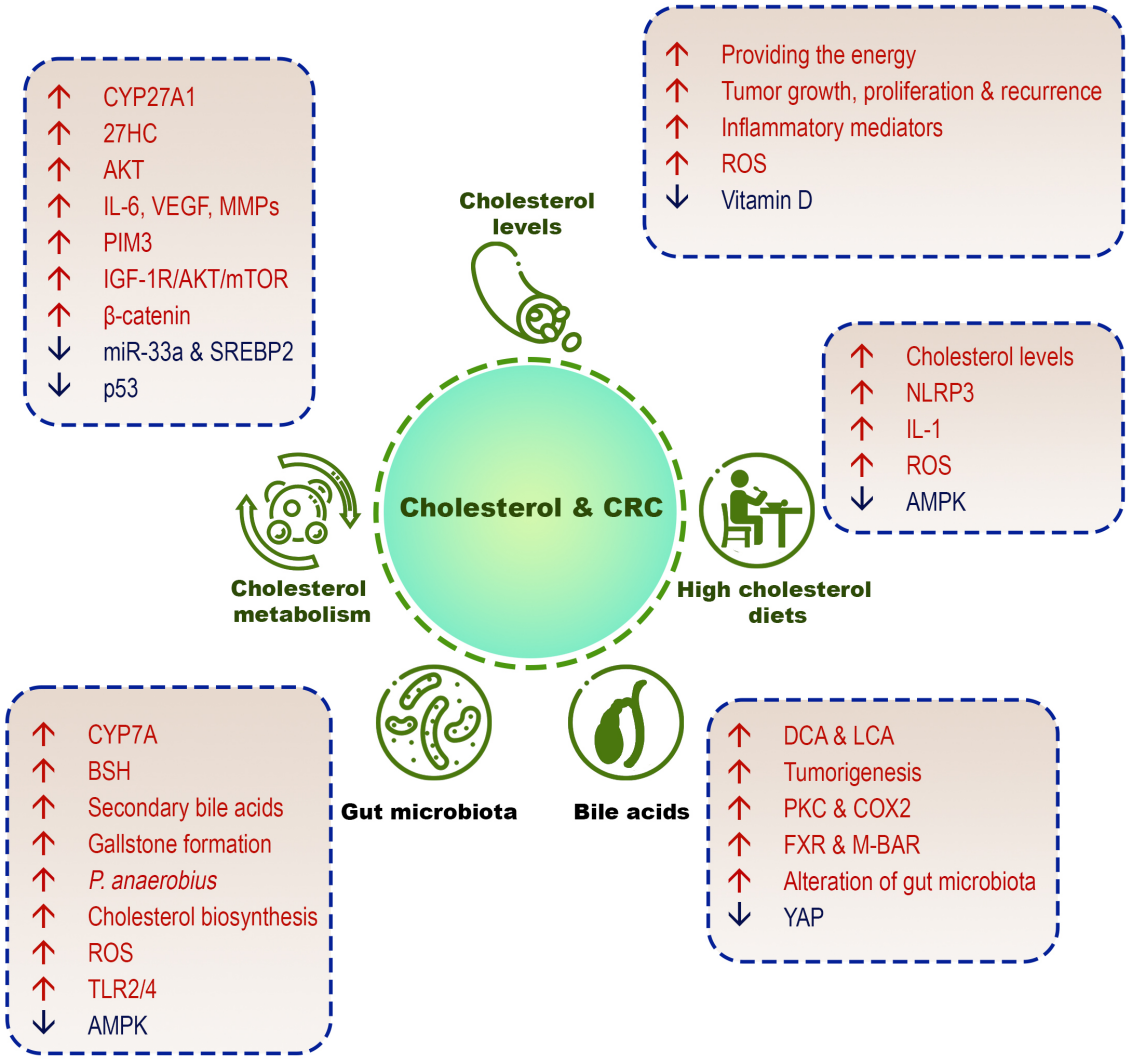
Different cholesterol-related factors in CRC development. High cholesterol diets, high serum cholesterol levels, cholesterol metabolism/biosynthesis, cholesterol-derived bile acids, and gut microbiome are involved in the pathogenesis of CRC.

This section discusses the different aspects of the role of cholesterol in increasing the incidence of CRC (**Figure 3**).

### 5.1 Diets and risk of colorectal cancer

Among the environmental factors that increase susceptibility to CRC, diet, environmental pollution, and physical activity are more significant (60–62). Highlighting the contribution of dietary cholesterol to CRC and underscoring the role of dietary-free cholesterol in its pathophysiology is crucial for a comprehensive understanding of the subject. Numerous studies have demonstrated the potential link between dietary cholesterol and CRC (63). A diet high in cholesterol has been associated with an increased risk of CRC development, with cholesterol-rich foods, such as red meat and high-fat dairy products, particularly implicated (64). Evidence demonstrated that tumor cells have a multifaceted relationship with cholesterol. They primarily synthesize their cholesterol through *de novo* synthesis. However, these cells can also obtain cholesterol from external sources by taking up exogenous free cholesterol and cholesterol esters from lipoproteins like HDL, LDL, and VLDL (65). Tumor cells can store excess cholesterol in lipid droplets as cholesterol esters to manage excess cholesterol and prevent potential toxicity. Furthermore, excess cholesterol can be either directly exported from the cell or converted into bile acids for eventual excretion. Additionally, cholesterol-bound fatty acids can be broken down through mitochondrial β-oxidation to produce energy, typically inside the mitochondria. This intricate interplay with cholesterol underlines its importance in cancer cells’ cellular processes and survival (65).

In Western diets, animal-related foods, such as red meat and egg, are the primary source of exogenous cholesterol and increase the risk of CRC (66). A recent study reported that red (in male sex and younger age cases) and processed meat (in older age) account for 1.77% and 1.18% of worldwide CRC mortality, respectively (67). However, regarding red and processed meat consumption and their association with CRC, the outcomes are contradictory, and some studies have reported a weak and non-significant relationship (68, 69).

Several studies indicate that unsaturated fat may contribute to CRC; however, only some of these investigations account for the differences in overall energy intake. After correction for increased calorie intake, it was concluded that there was little evidence for an increased risk associated with dietary fat after a meta-analysis of case-control studies. Increased calorie intake was also strongly associated with CRC risk (69, 70). It seems that CRC cannot be linked to a specific element in various diets, such as cholesterol or unsaturated fats. It has been shown that diets rich in cholesterol and saturated fats, such as Western diets, can induce CRC by increasing the production of reactive oxygen species in the bowel, inflammation, and mutagenesis (71).

On the other hand, low-fat and low-cholesterol diets, such as diets based on plant foods, can have a protective role (70). There is evidence that cholesterol-rich diets and those that would raise serum total cholesterol are associated with a higher risk of CRC; however, the association between total calorie intake, metabolic syndrome, and obesity appears to be more significant (11). Studies demonstrated that using a cholesterol-enriched diet for a long time could significantly induce chronic inflammatory responses and develop CRC. For instance, an animal model study reported that feeding with high-cholesterol diets and further producing cholesterol crystals could dysregulate inflammasomes through the activation of the NLR family pyrin domain containing 3 (NLRP3) and interleukin-1 beta (IL-1β) secretion, developing inflammatory bowel diseases (IBDs), and colitis-associated cancer (CAC). Crystal uptake and cathepsin B are responsible for the inactivation of the AMP-activated protein kinase (AMPK) pathway in macrophages and the overproduction of mitochondrial ROS, activating NLRP3 (72).

Moreover, adipose tissue, commonly known as fat tissue, is involved in developing CRC (27). Adipose tissue is an energy storage depot and functions as an active endocrine organ, releasing various adipokines and pro-inflammatory mediators (73). Higher levels of adipokines, such as leptin, and elevated production of inflammatory cytokines, like interleukin-6 (IL-6) and tumor necrosis factor-alpha (TNF-α), in the adipose tissue of people with obesity can contribute to chronic inflammation and insulin resistance. These factors are associated with an increased risk of CRC development through several mechanisms, including promoting cell proliferation, inhibiting apoptosis, and enhancing angiogenesis (74). In addition, adipose tissue can influence the gut microbiota pattern and their composition, which may further influence the risk of CRC (75). Consequently, the association between adipose tissue and CRC highlights the significance of preserving a healthy body weight and adipose tissue function to alleviate the risk of CRC.

### 5.2 Cholesterol levels and colorectal cancer

The correlation between high serum cholesterol levels and CRC has been established for several years. However, the advanced disease may cause a decline in serum cholesterol levels, which may explain why some early studies have shown an inverse association between serum cholesterol concentrations and the risk of CRC (12).

Since lipids may enhance tumor recurrence by providing the energy required for tumor cell growth proliferation, analysis of various lipid fractions and molecules that impact cholesterol metabolism could be beneficial. Recent studies showed that among total cholesterol, HDL, LDL, triglycerides, and apolipoproteins, serum lipid levels of triglycerides and LDL-C were not associated with CRC recurrence (76). However, another study reported that cholesterol homeostasis is switched to increase cholesterol biosynthesis because the relative abundance of non-cholesterol sterols (NCS), as cholesterol synthesis and absorption biomarkers, was amplified in HDL particles. These data indicate cholesterol precursors are overproduced in peripheral tissues (77). Molecular mechanisms have been proposed to explain the association between cholesterol metabolism and CRC, including fluctuations in the inflammatory responses and cytokine levels that affect the proliferation and apoptosis of CRC tumor cells, as well as a reduction in oxidative stress through the reduction of oxidized LDL (oLDL) (78, 79).

Nonetheless, analyzing insulin and inflammatory adipokines revealed that only insulin and leptin were significantly associated with the risk of CRC. According to the role of polyps in CRC, an investigation reported that low serum cholesterol was not linked to the development of CRC. However, smoking was identified as a potential confounding variable strongly related to the presence of polyps (80). Interestingly, a significant positive association exists between serum total cholesterol and vitamin D levels. Serum cholesterol levels can decrease vitamin D (1,25-dihydroxy-vitamin D3) and increase the risk of poorly differentiated CRC (81).

### 5.3 Association between bile acids, gut microbiota, and colorectal cancer

Evidence revealed a potential link between cholesterol metabolism and CRC through the association between bile acids and CRC. The basis for this association is the similarity in structure between bile acids and carcinogenic polycyclic aromatic hydrocarbons. As the secondary bile acids that are involved in tumorigenesis, deoxyxholic acid (DCA) and lithocholic acid (LCA) are formed by deconjugating primary bile acids by anaerobic bacterial flora (82, 83). In Western countries, it has been reported that the feces of individuals comprise more anaerobic bacteria responsible for deconjugating primary bile acids (84, 85). Higher fecal levels of secondary bile acids and cholecystectomy can also develop CRC (86). Secondary bile acids, via activating protein kinase C (PKC) and cyclooxygenase 2 (COX2) pathways, promote the growth, proliferation, and invasion of tumor cells in CRC (87). As discussed, SNPs in *CYP7A1*, which converts cholesterol to primary bile acids, could be associated with increased CRC risk (13). Following the deconjugating of bile acids by gut microbiota, diverse bile acid compounds can interact with host receptors, such as the farnesoid X receptor (FXR), a nuclear receptor activated by bile acids membrane-type receptor for bile acids (M-BAR) or TGR5. Moreover, modulation of the yes-associated protein (YAP)-associated pathway in intestinal epithelial cells by M-BAR induces CRC (88).

Interestingly, recent studies revealed that serum bile pigment bilirubin concentrations, through altering gut microbiota patterns, are inversely correlated to gut inflammation and the risk of CRC. Furthermore, fat-mediated changes in the gut microbiota can link the metabolism of bile acids to the risk of CRC risk and colonic tumorigenesis (89). Therefore, bile pigments and bile acids have multiple impacts on gut microbiota composition, which may induce or inhibit the development of CRC risk (88). Gut microbiota is essential in preserving human health in various parts (90). As discussed, the pattern of primary bile acids as endogenous cholesterol-derived molecules can be converted to secondary bile acids by the gut microbiota by releasing critical enzymes, such as bile salt hydrolases (BSH) and CYP7A (91–93). Following these enzymes’ activity, the gut microbiome’s pattern, bile acids profile, and metabolism are modified. Gallstone formation is another consequence of changing bile acids profile and metabolism (93). Secondary bile acids can act as signaling molecules and regulate host metabolic phenomena. In patients with longtime asymptomatic gallstones, the risk of CRC was significantly increased, which may be correlated with the modified gut microbiome and bile acids metabolism (94).

Metagenome sequencing revealed that stool specimens from CRC patients or colorectal adenoma showed more *Peptostreptococcus anaerobius* than subjects without CRC. An animal study showed that intestinal dysplasia occurred more in bacteria-depleted mice exposed to azoxymethane and *P. anaerobius* than in mice exposed to only azoxymethane. Furthermore, *P. anaerobius* tended to colonize the colon and induce the growth and proliferation of the epithelial cells in the colon. Interestingly, *P. anaerobius* could upregulate the genes’ expression in regulating cholesterol biosynthesis, the AMP-activated protein kinase (AMPK) pathway, and toll-like receptor (TLR) signaling. *P.anaerobius* activates SREBP2 and increases total cholesterol levels. It also interacted with TLR2 and TLR4 to raise intracellular ROS levels, promoting cholesterol biosynthesis and cell proliferation (95).

### 5.4 Cholesterol metabolism and colorectal cancer

The cholesterol metabolism, via acting on innate and adaptive immune cells, plays a significant role in modulating antitumor responses. In this regard, tumor-associated macrophages (TAMs) can deplete cholesterol in some human malignancies (96). Additionally, macrophages with a morphology are correlated to increased cholesterol metabolism, liver metastasis, and poor survival in patients with CRC (97). Interestingly, TAMs can contribute to the cholesterol supply for cancer cells through a process known as cholesterol efflux and transport. In the TME, TAMs often undergo a phenotypic switch towards a pro-tumoral, M2-like phenotype, which can facilitate cholesterol transfer to tumor cells (96). These M2-like TAMs express high levels of scavenger receptors, such as CD36, which can bind to and uptake excess cholesterol from the tumor milieu (98). The cholesterol is then transported within TAMs through intracellular lipid droplets and may be transferred to cancer cells via cholesterol-rich particles called lipid rafts or through direct cell-to-cell contact. This cholesterol supply can promote the growth and survival of cancer cells (99).

CYP27A1 overexpression is associated with poor prognosis in CRC (100). It has been demonstrated that CYP27A1 expressed in various tissues produces 27-hydroxycholesterol (27HC), a rich oxysterol in the blood and plasma membrane (101). Despite the controversial role of 27HC in human tumors, an investigation has reported that 27HC can induce AKT activation and the secretion of IL-6, vascular endothelial growth factor (VEGF), and matrix metalloproteinases (MMPs), resulting in CRC development. In contrast, another study disclosed that 27HC suppressed AKT activation and inhibited the proliferation of tumor cells in CRC *in vitro* (102).

miRNAs (miRs) are non-coding RNAs regulating gene expression at the post-transcriptional level. miRs are also implicated in various biological functions of organisms. The overexpression of multiple miRNAs has been reported in human malignancies, which were strictly associated with the occurrence and expansion of tumors (103, 104). It has been shown that miR-33a can target Pim-3 proto-oncogene and serine/threonine kinase (*PIM3*). In addition, miR-33a is an essential factor in cholesterol metabolism. miR-33a is also overexpressed in several human cancers. Cholesterol can inhibit the expression of miR-33a and SREBP2. Moreover, cholesterol treatment could upregulate PIM3, promoting tumor cell proliferation and inhibiting apoptosis via phosphorylation of Bad protein, p21, and p27. Consequently, it is possible that cholesterol regulates the development of CRC via the miR-33a/PIM3 pathway, and it could be a potential therapeutic target for treating CRC (105).

Squalene epoxidase (SQLE) is also a rate-limiting enzyme involved in cholesterol biosynthesis and is known as a proto-oncogene (106). High cholesterol concentrations can lead to the degradation of SQLE. Furthermore, reduced SQLE levels via cholesterol accumulation, activating the β-catenin oncogenic pathway, and inhibiting p53 can be associated with CRC. These outcomes show that SQLE is a critical regulator in the development of CRC (107). It has been found that the insulin-like growth factor receptor (IGF-1R)/AKT/mTOR and the mevalonate-isoprenoid biosynthesis pathways were upregulated in CRC stem cells (CSCs), inducing tumor cell growth and proliferation. Targeting these pathways would probably be helpful in CRC treatment (108).

## 6 Therapeutic approaches

Until today, various treatment methods have been investigated for treating CRC. For instance, conventional therapies, such as surgery, chemotherapy, radiation therapy, targeted therapy, and immunotherapy, have been used to treat patients with CRC (109, 110). Although the results of these treatment methods are not satisfactory and the discovery of novel or complementary approaches can help to increase the effectiveness of the treatment. In this section, we have summarized available cholesterol-related therapeutic tactics for treating CRC (**Table 1**).

**Table 1:** Cholesterol-related therapies for CRC treatment.

Approach	Specifications	Mechainsm	Ref
**Minor modifications in Western diets**	• Substituting fats with carbohydrates or saturated fats with polyunsaturated fats as well as consuming more fruits and fiber	• Could not significantly reduce the risk of CRC	(111, 112)
**Controlled diet**	• Fewer calories and containing more fruits and vegetables and less fat and red meat	• Protective role against CRC	(11, 113)
**Fasting**	• Stop eating completely for 12-24 h	• **↑** The expression of *FDFT1* • **↓** AKT/mTOR/HIF1α pathways • **↓** Tumor cell growth and progression	(114)
**Statins**	• Inhibition of HMG-CoA reductase	• **↓** Isoprenoid production • ↓ Tumor growth & proliferation • **↑** BMP pathway • ↓ KRAS prenylation • **↑** Tregs • ↓ Angiogenesis	(115, 116) (117, 118) (119)
**Simvastatin + anti-immune checkpoint therapy**	• Inhibition of HMG-CoA reductase & PD-L1	• ↓ PD-L1 expression • **↑** Anti-tumor immune responses • ↓ lncRNA SNHG29 • ↓ CRC liver metastasis • ↓ Cholesterol biosynthesis	(120) (121)
**Nystatin with avasimibe**	• Inhibition of HMG-CoA reductase • SOAT1 inhibitor	• ↓ YAP expression • ↓ The viability of tumor cells *in vitro* and *in vivo*	(122)
**Fibrates**	• Activation of PPAR-α	• ↓ Lipid levels • ↓ The risk and development of CRC	(123, 124)
**BPH1222 and zoledronic acid**	• Interfere with the mevalonate pathway, squalene synthase, and cholesterol biosynthesis	• ↓ Cell viability dose-dependently *in vitro* • **↑** Tumor cells in the S and sub-G1 phases • Changing the phosphorylation of ERK and S6 • ↓ Tumor growth in *KRAS* mutant xenograft mouse	(8) (125)
**T0901317**	• LXR inhibition	• ↓ β-catenin transcriptional activity in HCT116 cells • ↓ SREBP pathway • ↓ Tumor growth	(126)
**GW3965**	• LXR inhibition	• ↓ SREBP pathway • ↓ Tumor growth proliferation	(127)
**R048-8071**	• Lanosterol synthase inhibiton	• ↓ The phosphorylation of AKT • ↓ Tumor growth and metastasis	(128)
**Docosahexaenoic acid & ursodeoxycholic acid**	• Inhibition of HMG-CoA reductase, NPC1, and SREBF2	• ↓ Tumor growth proliferation	(129, 130)
**TASIN**	• Selective toxic compounds for *APC* mutations of CRC	• ↓ EBP • ↓ DHCR7 • ↓ DHCR24 • ↓ Post-squalene cholesterol synthesis pathway • Depleting downstream sterols • ↓ Tumor growth proliferation	(131)
**Terbinafine**	• SQLE inhibitor	• ↓ CRC cell growth and proliferation in organoids and xenograft animal models • ↓ Calcitriol and activating the CYP24A1-mediated MAPK pathway • ↓ Tumor growth proliferation	(132)
**Metformin**	• AMPK activator	• ↓ CSCs • ↓ Mevalonate pathway and cholesterol biosynthesis, • **↑** p-AMPK • ↓ mTOR expression	(133)
**C1 & C2**	• Binding to β-catenin, which inhibited the formation of β-catenin/BCL9 complex	• ↓Wnt activity • ↓ The expression of the Wnt/β-catenin signature • Interrupted cholesterol homeostasis • ↓ Tumor cell growth and proliferation	(134)
**Knockdown of SREBP1 or SREBP2**	• SREBP-dependent metabolic regulation	• ↓ Glycolysis • ↓ Mitochondrial respiration, and fatty acid oxidation • Cell metabolism alteration, reduced fatty acid levels • ↓ Cell proliferation • ↓ Tumor spheroid formation • ↓ Tumor growth in xenograft models of colon cancer • ↓ The expression of cancer stem cell-associated genes	(45)
**Aptamer PL1 + siRNA**	• Aptamer PL1 specifically binds to PD-L1 • siRNA that targets PCSK9	• **↑** The effectiveness of anti-immune checkpoint therapy • **↑** The expression of IFN-γ and granzyme B • ↓ Tumor growth proliferation	(135)
**HCE**	• Antineoplastic and anti-inflammatory properties • Cholesterol biosynthesis inhibition	• Selective cytotoxic effect of HCE on tumor cells • ↓ The Notch pathway • ↓ Cholesterol biosynthesis • ↓ Tumor growth proliferation	(136)
**Curcumin**	• Via Ca^2+^/PPARγ/SP-1/SREBP-2/NPC1L1 signaling and TRPA1	• ↓ Tumor cell proliferation • ↓ Cholesterol absorption in Caco-2 cells • Benefit the primary CRC prevention	(137)
**FE & HFE** **(Saponins or sapogenins)**	• Inhibitio of lipid metabolism	• ↓ Aerobic glycolysis and mitochondrial oxidative phosphorylation • ↓ The expression of *TYMS1* and *TK1* • ↓ Intracellular lipid concentrations.	(138)

↑, increase; ↓, decrease.

### 6.1 Dietary prevention of colorectal cancer

Since Western diets are associated with a high incidence of CRC, nutrition interventions may help prevent the disease. Previous studies showed that even minor modifications in Western diets, including substituting fats with carbohydrates or saturated fats with polyunsaturated fats as well as consuming more fruits and fiber, could not significantly reduce the risk of CRC (111, 112). In these studies, the total calorie intake was notably the same before and after the modifications (139). Since obesity, inactivity, and consumption of red meat have been introduced as the most critical environmental factors that increase the risk of CRC, it appears that diets with fewer calories and containing more fruits and vegetables and less fat and red meat can have a protective role against CRC (11, 113). An investigation demonstrated that fasting could affect glucose and cholesterol metabolism in CRC. Fasting also upregulated the expression of the farnesyl-diphosphate farnesyltransferase 1 (*FDFT1*) as a tumor suppressor and cholesterogenic gene. *FDFT1* regulates the pathways of AKT/mTOR/hypoxia-inducible factor 1 α (HIF1α). Therefore, decreased expression of *FDFT1* is associated with tumor cell growth and progression and poor prognosis in CRC (114). However, since this gene plays a role in synthesizing sterols and cholesterol, its upregulation may increase cholesterol levels and tumorigenesis. As a result, more studies are required to clarify the role of this gene in cancer.

### 6.2 Targeting cholesterol biosynthesis, metabolism, and absorption

#### 6.2.1 Statins

Statins are HMG-CoA reductase inhibitors, a class of lipid-lowering drugs used in several cancers, such as esophageal, gastric, hepatic, and prostatic cancer (115). Statins can suppress tumor cell growth, invasion, and metastasis via inhibiting isoprenoid production because they are essential for post-translational protein modifications (115, 116). Based on available knowledge, patients with inflammatory bowel disease (IBD) are more susceptible to developing CRC. It has been reported that treating IBD patients with statins reduced the risk of CRC (140). Other clinical studies showed that patients receiving statins for a long time have a 47% lower risk of CRC than non-statin subjects (129). A large population-based cohort also reported that treating CRC patients with statins prolonged the survival rate of the studied patients (141). Statins may activate the bone morphogenetic protein (BMP) pathway because these drugs are more effective in SMAD family member 4 (SMAD4)-expressing cancers but not *KRAS* mutant tumors (117, 118). In CRC, statins are thought to act via changes in KRAS prenylation. However, this would be expected to alter CRC proportion via induction of mutations in *KRAS* (142). Evaluation of the effects of statins on immune system components in CRC showed that the frequency and infiltration of regulatory T cells (Tregs) into the CRC TME significantly increased following statin therapy in advanced stages of CRC. In the early stages of CRC, statins inhibited angiogenesis, but they could not considerably affect transforming growth factor-beta 1 (TGF-β_1_) levels in tumor tissue. This study suggested that infiltrating Tregs into the TME by statins might decrease tumor aggressiveness in the advanced stages of CRC (119). Regarding the dual role of Tregs in cancer, these results could be controversial and require further investigations based on the types and stages of cancers (143).

On the other hand, several meta-analyses disclosed that statins had little impact (about 10%) on reducing the risk of CRC (144). However, because CRC is a heterogeneous tumor, finding an effective treatment in all cases is challenging. Statins were more effective in treating CRC than lipid-lowering agents, such as fibrates. In this regard, an investigation showed that fibrate administration could not affect CRC incidence, whereas statins were more efficient (145). Fibrates decrease lipid levels by activating peroxisome proliferator-activated receptor alpha (PPAR-α) and, in this way, can reduce the risk and development of CRC (123, 124). Therefore, reducing the risk of CRC by statins and fibrates as modificators of cholesterol metabolism may depend on their impacts on KRAS prenylation or the PPAR-α pathway.

One of the most important challenges in cancer therapy is the immunosuppressive milieu in the TME. Infiltration of immunosuppressive cells, as well as expression of inhibitory molecules, such as programmed death-1 (PD-1), programmed death ligand 1 (PD-L1), and V-domain immunoglobulin suppressor of T-cell activation (VISTA), could modulate antitumor immune responses (146). Immune checkpoint inhibitors, including anti-PD-1 and anti-PD-L1 monoclonal antibodies (mAbs), are a promising therapeutic approach for cancer therapy. Nevertheless, early clinical trials revealed ineffective immune checkpoint inhibitors in CRCs. An investigation reported that simvastatin could suppress PD-L1 expression and promote anti-tumor immune response by downregulating lncRNA SNHG29 expression. SNHG29 targets YAP and inhibits its phosphorylation and ubiquitination, accelerating the decrease in the expression of PD-L1 transcript. Therefore, the administration of simvastatin can suppress lncRNA SNHG29-mediated YAP activation and induce anti-tumor immune responses by hindering the expression of PD-L1 (120). Simvastatin can also inhibit CRC liver metastasis by reducing cholesterol biosynthesis because hepatocyte growth factor (HGF) released from the liver induces SREBP2 and activates the c-Met/PI3K/AKT/mTOR axis in tumor cells to increase the cholesterol biosynthesis (121).

Sterol O-acyltransferase 1 (SOAT1) converts intracellular free cholesterol to cholesteryl ester (147). These cholesteryl esters can be stored as lipid droplets via SOAT1-mediated esterification. It has been shown that SOAT1 targeting could upregulate the expression of YAP through increasing cellular cholesterol concentrations in colon cancer cells. In addition, under SOAT1 suppression, sequestrating cholesterol by nystatin significantly repressed YAP expression. Combining nystatin with avasimibe as a SOAT1 inhibitor could reduce the viability of tumor cells *in vitro* and *in vivo* (122). Together, statins can be somewhat effective in treating CRC by disrupting cholesterol metabolism and biosynthesis. However, tumor profiling may lead to recognizing a molecular subtype of tumors that are more sensitive to statins to increase targeted therapies’ effectiveness.

#### 6.2.2 Bisphosphonates

Bisphosphonates used to treat bone disorders can also interfere with the mevalonate pathway, squalene synthase, and cholesterol biosynthesis (125). In CRC, long-administrating bisphosphonates may have a protective role and reduce the risk of CRC; however, several studies believe these drugs are not more effective than statins in treating CRC (148, 149). Recently, the antitumor effects on RAS-mediated signalization of lipophilic bisphosphonate (BPH1222) and zoledronic acid were investigated on several human CRC cell lines. The findings showed that these bisphosphonates could decrease cell viability dose-dependently *in vitro*. In addition, BPH1222 and zoledronic acid affected the cell cycle by increasing the frequency of tumor cells in the S and sub-G1 phases. These drugs also changed the phosphorylation of ERK and S6 proteins. Further studies on *KRAS* mutant xenograft mouse model showed an inhibitory effect on the growth of treated CRC tumor cells with the lipophilic bisphosphonates (8). Therefore, even though these drugs are not designed to treat cancer, they can inhibit the growth of tumor cells in different ways and can also be used to treat *KRAS* mutants CRC.

#### 6.2.3 LXR targeting in colorectal cancer

The nuclear receptors liver-X-receptors (LXRs) play a pivotal role in regulating the homeostasis of lipids and intracellular cholesterol (150). Oxysterols are ligands of LXRs and suppress the SREBP pathway and cell proliferation. It has been revealed that 27HC (an oxysterol) treatment inhibits cell proliferation in CRC tumor cells; however, this inhibitory effect is mediated by reducing the phosphorylation of AKT compared with activating LXR (102). Other oxysterols, including 5α-cholestane-3β,6β-diol, 7-ketocholesterol, and cholestane-3β-5α-6β-triol exert their inhibitory effects through arrest in the cell-cycle progression and inducing apoptosis in tumor cells (151). However, according to the tumor context, oxysterols can play a dual-edged sword in cancer (129). Moreover, LXRα activation can affect lipid metabolic pathways and increase cholesterol efflux through some membrane proteins, such as ATP binding cassette subfamily A member 1 (ABCA1) and ABCG5/G8, in the intestine (126). Upregulation of the activated LXRα also can repress CRC cell proliferation *in vitro* and *in vivo*. Consequently, designing agonists of LXR might be a hypothetical therapeutic method for CRC treatment (152). As an LXR agonist, T0901317 can suppress β-catenin transcriptional activity in HCT116 cell lines (colon cancer) *in vitro* (126). Additionally, another investigation demonstrated that the upregulation of the nuclear receptor subfamily 1 group H member 3 (*NR1H3*) gene, which encodes LXRα, could induce inhibitory effects of GW3965 as an LXRs agonist on the proliferation of tumor cells in CRC. *NR1H3* is also able to suppress the activity of the epidermal growth factor receptor (EGFR) promoter, reducing tumor cell growth and proliferation (127).

#### 6.2.4 Other effective drugs

R048-8071 or (4-bromophenyl)[2-fluoro-4-[[6-(methyl-2-propenylamino)hexyl]oxy]phenyl]-methanone is a subclass of benzophenones and an oxidosqualene cyclase (OSC) inhibitor. OSC converts 2,3-oxidosqualene to lanosterol, and its inhibition by Ro 48-8071 leads to inhibit the growth, migration, and metastasis of tumor cells in CRC (153). Using R048-8071 also can reduce cell proliferation and induce tumor cell apoptosis (129). Targeting lanosterol synthase (LSS) by R048–8071 represses the phosphorylation of AKT and inhibits the growth and metastasis of both pancreatic cancer and CRC (128).

Since the endoplasmic reticulum (ER) is the primary site for cholesterol biosynthesis, oxidation, and esterification, ER cholesterol concentrations are associated with the activation of SREBP2. Hence, ER stress may affect cholesterol metabolism. It has been reported that the administration of docosahexaenoic acid enhanced ER stress in the CRC cell line, SW620. Docosahexaenoic acid can upregulate the expression of key genes in cholesterol metabolism, such as HMG-CoA reductase, NPC intracellular cholesterol transporter 1 (NPC1), and SREBF2 (129). Ursodeoxycholic acid also could be an effective and well-tolerated chemopreventative method to treat CRC (130). However, it has been reported that a high dose of ursodeoxycholic acid in primary sclerosing cholangitis leads to an increase in the risk and development of CRC, and this challenge has limited its application (154).

Truncated APC-selective inhibitors (TASIN) are selective toxic compounds for *APC* mutations of CRC. An investigation found that TASINs could suppress the emopamil binding protein (EBP), 7-dehydrocholesterol reductase (DHCR7), and DHCR24 enzymes involved in the post-squalene cholesterol synthesis pathway. In spite of the fact that all three of these enzymes are necessary for cholesterol biosynthesis, inhibiting EBP led to the death of tumor cells by depleting downstream sterols (131).

SQLE can affect cholesterol biosynthesis, and it has been revealed that this enzyme is upregulated in CRC *in vitro* and *in vivo* and is associated with poor prognosis in patients with CRC. Furthermore, SQLE inhibition reduces the calcitriol (the active form of vitamin D3) levels and CYP24A1. In addition, SQLE inhibition can increase intracellular Ca^2+^ levels. Afterward, the MAPK pathway is suppressed, inhibiting CRC cell growth and proliferation (132). One of these SQLE inhibitors is terbinafine which can suppress CRC cell growth and proliferation in organoids and xenograft animal models (132). Therefore, by accumulating calcitriol and activating the CYP24A1-mediated MAPK pathway, SQLE promotes CRC, suggesting that SQLE could be a probable therapeutic target for CRC treatment.

Metformin is an AMPK activator and inhibits cancer stem cells (CSCs) in several malignancies. A study showed that metformin, via affecting the mevalonate pathway and cholesterol biosynthesis, could reduce the CSC population in both DLD-1 and HT29 CRC cell lines. This study reported that metformin therapy could amplify p-AMPK and decrease mTOR expression. Moreover, the mevalonate addition reversed these suppressive effects. These findings indicate that in CRC, metformin suppresses CSC through AMPK activation and mevalonate pathway inhibition, which is related to AMPK activation (133). A recent study also showed that dysregulation of the Wnt/β-catenin pathway could be involved in the pathogenesis of CRC. This study used a novel compound capable of binding to β-catenin, which inhibited the formation of β-catenin/BCL9 complex in CRC cell lines. The compound also suppressed Wnt activity, reduced the expression of the Wnt/β-catenin signature, interrupted cholesterol homeostasis, and remarkably decreased tumor cell growth and proliferation in CRC *in vitro* and *in vivo* (134). Therefore, the Wnt/β-catenin could be involved in cholesterol metabolism, and targeting this pathway can help treat CRC by inhibiting the production of cholesterol and the growth of cancer cells.

#### 6.2.5 Targeting essential genes in cholesterol metabolism and biosynthesis

Several studies on various human malignancies demonstrated that Krüppel-like factors (KLFs) are involved in developing cancer. KLF13 transcriptionally suppresses HMG-CoA synthase and cholesterol biosynthesis. In CRC, KLF13 is downregulated, and functional experiments revealed that knockdown of the KLF13 induced the growth, proliferation, and colony formation in CRC cell lines (HT-29 and HCT116). Moreover, the upregulation of KLF13 could induce cell cycle arrest at G0/G1, reduce 5-ethynyl-2’-deoxyuridine (EdU) incorporation, and suppress HCT116 cell growth in nude mice. Additionally, the knockdown of HMG-CoA synthase repressed cholesterol biosynthesis and the proliferation of silenced KLF13 tumor cells in CRC (136). Accordingly, KLF13, as a tumor suppressor factor, negatively regulates the HMG-CoA synthase-mediated cholesterol biosynthesis in CRC, and its knockdown cannot benefit the treatment.

Another investigation reported that the knockdown of SREBP1 or SREBP2 through decreasing glycolysis, mitochondrial respiration, fatty acid oxidation, and cell metabolism alteration reduced fatty acid levels. Furthermore, cell proliferation and the ability of tumor spheroid formation by cancer cells were significantly reduced following SREBP1/SREBP2 knockdown. In addition, the knockdown of SREBP1/SREBP2 could suppress tumor growth in xenograft models of colon cancer and downregulate the expression of cancer stem cell-associated genes. As a result of these findings, the molecular basis of SREBP-dependent metabolic regulation has been established, and targeting lipid biosynthesis may prove to be a promising therapeutic approach in colon cancer (45).

PCSK9 can bind to LDL receptors, resulting in their degradation in the liver. It has been revealed that PCSK9 inhibition increases LDL receptors and decreases blood levels of LDL (155). Upregulation of PCSK9 is associated with an unpromising survival rate in *APC*/*KRAS*-mutant CRC patients. Moreover, depleting PCSK9 inhibits the growth of *APC*/*KRAS*-mutant CRC cells *in vitro* and *in vivo*. However, overexpression of PCSK9 is associated with tumorigenesis. Interestingly, PCSK9 decreases cholesterol uptake while promoting *de novo* synthesis and geranylgeranyl diphosphate (GGPP) accumulation. GGPP, a critical PCSK9 downstream metabolite, can activate the KRAS/MEK/ERK pathway. It has been revealed that PCSK9 inhibitors, especially in combination with statins, repress the growth of *APC/KRAS*-mutant CRC cells. These data suggest that PCSK9 induces *APC*/*KRAS*-mutant CRC through the GGPP-KRAS/MEK/ERK pathway, and its targeting may be a therapeutic method in treating CRC (15, 156). Recently, a study designed an aptamer PL1 which specifically binds to PD-L1. This study showed that combining aptamer PL1 and a siRNA that targets PCSK9 synergistically increased the effectiveness of anti-immune checkpoint therapy via upregulating the expression of interferon-gamma (IFN-γ) and granzyme B (135).

#### 6.2.6 Natural compounds

It has been demonstrated experimentally that herbal extracts and isolated herbal compounds such as curcumin, matairesinol, and resveratrol can reduce resistance to cancer therapies and exert chemoprotective effects when combined with antitumor drugs (157). Hibiscus. syriacus callus extract (HCE) is one of these natural compounds with antineoplastic and anti-inflammatory properties. An investigation explored HCE effects on CRC cell lines (HT-29) and thymus-deficient mice bearing xenografts. Outcomes obtained from cell viability and colony formation assays showed a notable and selective cytotoxic effect of HCE on tumor cells. HCE cytotoxic effects were associated with inhibiting the Notch pathway, positively contributing to cholesterol biosynthesis *in vitro* and *in vivo* without systemic toxicity (136). Moreover, it has been shown that curcumin via the Ca^2+^/PPARγ/SP-1/SREBP-2/NPC1L1 signaling and also through stimulating the transient receptor potential ankyrin 1 (TRPA1) can suppress tumor cell proliferation and reduce cholesterol absorption in Caco-2 cells. These data indicate that curcumin as a dietary supplement may benefit primary CRC prevention (137).

Saponins or sapogenins)aglycone form of saponins) are known as bioactive agents with anticancer features (158). It is widely recognized that metabolic reprogramming is a hallmark of cancer and that altered lipid metabolism, hypercholesterolemia, increased aerobic glycolysis, and glutaminolysis are essential to cancer development and progression (159). Using saponin-rich extracts from fenugreek (FE) and its hydrolyzed extract (HFE) as sapogenin-rich extracts showed that HFE inhibited aerobic glycolysis and mitochondrial oxidative phosphorylation. The expression of thymidylate synthase (*TYMS1*) and thymidine kinase 1 (*TK1*) also downregulate following combining treatment with FE and drug 5-fluorouracil (5-FU). Additionally, HFE could inhibit lipid metabolism targets, reducing intracellular lipid concentrations (138). Thus, saponins and sapogenins alone or combined with chemotherapeutic agents may reduce tumor metabolism reprogramming and inhibit tumor cell growth and progression.

## 7 Challenges and limitations of therapeutic strategies

The diverse therapeutic approaches discussed for treating CRC offer promise and challenges. While non-invasive and cost-effective, dietary interventions face limitations in consistently producing significant reductions in CRC risk (160, 161). Statins, known for their potential to inhibit cholesterol biosynthesis and impact immune responses, have demonstrated promise in reducing CRC risk and improving survival rates in clinical studies, yet their effectiveness remains a topic of debate (144). Originally designed for bone disorders, bisphosphonates may not be as potent in CRC treatment as statins, highlighting their primary role in non-cancer therapy (162, 163).

Moreover, targeting LXRs appears advantageous due to their impact on lipid metabolism and cholesterol efflux, but the complex dual role of oxysterols in cancer and treatment outcomes necessitates further investigation (164). Strategies focused on regulating essential genes, such as *KLF13* and *SREBP1*/*SREBP2*, can suppress cell proliferation and tumor growth but may only universally apply to some molecular subtypes of CRC (45, 165). Natural compounds like curcumin and saponins show potential in reducing treatment resistance and exerting chemoprotective effects when combined with traditional therapies, but their optimal doses and potential side effects require further research (166). The major challenge in CRC treatment lies in its heterogeneity, necessitating personalized approaches, and the complex immunosuppressive TME, which complicates the effectiveness of immune checkpoint inhibitors (167). As the interactions between cholesterol metabolism, cancer, and therapy are intricate, thorough investigation and clinical trials are imperative to address these challenges and maximize the utility of cholesterol-related tactics in CRC treatment.

## 8 Concluding remarks and future directions

Almost all therapeutic methods and anticancer drugs have advantages and disadvantages mentioned in various studies. In the case of cholesterol-lowering drugs, in addition to the different side effects observed, other items should be considered. For instance, tumor cells can categorize as statin-sensitive and statin-resistant cells. Therefore, statins could not be effective in all cancers. To overcome this challenge, combining statins with polyamine metabolism inhibitors, purine metabolism inhibitors, glycolytic system inhibitors, and pentose phosphate pathway inhibitors may improve the anticancer impacts of statins (168). Another example is metformin, which in addition to its remarkable anticancer properties, one of the significant challenges of its prescribing in cancer therapy is fast renal clearance (169). The use of drugs that intervene in the biosynthesis and metabolism of cholesterol in treating CRC has resulted in low effectiveness, which can be caused by different mutants or non-responder tumor cells to these compounds.

Additionally, the inhibitory tumor microenvironment (TME), tumor escape mechanisms, chemoresistance, hypoxic condition, infiltration of immunosuppressive cells, and inhibitory molecules are considered fundamental challenges in treating all solid tumors, such as CRC (170–172). On the other hand, considering cholesterol is an essential precursor for synthesizing several hormones and cell components, how much does its inhibition to reduce the growth and development of tumor cells affect normal cells and physiological mechanisms? It is considered a fundamental question in cholesterol targeting.

On the other hand, regarding the unique features of cholesterol, it can be used in nanosystems to increase the efficiency of cancer therapy. For instance, a cholesterol-coated PLGA nanoparticle has been designed to improve the encapsulation and delivery of oxaliplatin and retinoic acid in CRC. The findings showed that the cell viability and proliferation of CRC cell lines decreased following using this nanosystem (173).

Generally, it can be proposed that the synthesis and metabolism of cholesterol and the components involved in these pathways can be good targets for treatment. Although monotherapy may not significantly affect cancers with high heterogeneity, such as CRC, their combination with other anticancer approaches may be beneficial.

## Author contributions

XH: Writing – original draft. HL: Writing – original draft. KJ: Writing – original draft, Writing – review & editing. FL: Writing – original draft, Writing – review & editing.
